# Imperfect adherence in real life: a prevention‐effective perspective on adherence to daily and event‐driven HIV pre‐exposure prophylaxis among men who have sex with men – a prospective cohort study in Taiwan

**DOI:** 10.1002/jia2.25733

**Published:** 2021-05-20

**Authors:** Huei‐Jiuan Wu, Stephane Wen‐Wei Ku, Howard H. Chang, Chia‐Wen Li, Nai‐Ying Ko, Carol Strong

**Affiliations:** ^1^ Department of Public Health College of Medicine National Cheng Kung University Tainan Taiwan; ^2^ The Kirby Institute UNSW Sydney New South Wales Australia; ^3^ Division of Infectious Diseases Department of Medicine Taipei Veterans General Hospital Taiwan; ^4^ Division of Infectious Diseases Department of Medicine Taipei City Hospital Renai Branch Taiwan; ^5^ Department of Biostatistics and Bioinformatics Emory University Atlanta GA USA; ^6^ Division of Infectious Diseases Department of Internal Medicine National Cheng Kung University Hospital College of Medicine National Cheng Kung University Tainan Taiwan; ^7^ Department of Nursing College of Medicine National Cheng Kung University Tainan Taiwan

**Keywords:** HIV, PrEP, pre‐exposure prophylaxis, MSM, adherence, event‐driven PrEP

## Abstract

**Introduction:**

Both daily and event‐driven (ED) pre‐exposure prophylaxis (PrEP) have been demonstrated to be highly effective among men who have sex with men (MSM). Prevention‐effective adherence proposes that PrEP adherence should be aligned with the risk of HIV, which could be applied to both daily and ED PrEP adherence measurement. The objective of this study was to describe the relationship between the use of PrEP and sex events among the MSM PrEP users and identify factors associated with adherence among daily and ED MSM PrEP users.

**Methods:**

A multicentre, observational, prospective cohort study was conducted at three hospital‐based clinics in three urban cities of Taiwan from January 2018 to December 2019. MSM ages 18 years or older – at high risk of HIV acquisition and taking PrEP during the study period – were included in the analysis. MSM PrEP users were allowed to choose between daily and ED PrEP based on their preference. Data on sociodemographic characteristics, mental health, sexual behaviours, substance use and PrEP‐taking behaviours were collected at each visit.

**Results:**

A total of 374 MSM were included in the analysis with 1,054 visits. More than half (56%) of the PrEP users chose ED at the baseline and 150 regimen switches were reported by 21% of the participants. There was only one seroconversion documented during the study period. Most (84.2%) of the MSM PrEP users were able to adhere to PrEP during the most recent anal intercourse in the past one month. Among ED PrEP users with suboptimal adherence, the majority (81.9%) missed the pre‐coital dose. In the multivariable analysis, we found that participants who switched from daily to an ED dosing regimen were associated with poorer adherence to PrEP.

**Conclusions:**

A high level of PrEP adherence was observed among the majority of MSM in a real‐world setting. On the other hand, Taiwanese MSM switching from daily to ED dosing regimens were less likely to adhere to PrEP, suggesting that novel approaches focusing on a dosing switch would be necessary for MSM to improve their adherence to PrEP.

## 1 INTRODUCTION

Both daily and event‐driven (ED) pre‐exposure prophylaxis (PrEP) have been demonstrated to be highly effective to prevent HIV infections among men who have sex with men (MSM) [1, 2, 3]. Such high efficacy of ED PrEP indicates that PrEP provides protection even if taken less frequently than daily, depending on the adherence pattern to the timing of HIV exposures for MSM [2, 4, 5, 6]. Adherence to PrEP has been crucial for HIV prevention, especially for daily dosing regimens [7, 8, 9]. On the other hand, unlike daily PrEP, adhering to ED PrEP was defined as taking PrEP before, during, and after the sexual intercourse [2]. Prevention‐effective adherence is a recent paradigm that proposes that PrEP adherence should be aligned with the risk of HIV rather than taking it regularly regardless of the risk of HIV and shares similarities with ED PrEP adherence [10].

When measuring prevention‐effective adherence, we need a detailed understanding of when the sex event occurred, the number of pills taken and other HIV prevention tools used simultaneously. The need for this information increases the complexity of PrEP adherence measurement since sex events are difficult to measure and rely heavily on self‐report, which potentially has recall and social‐desirability biases [11, 12, 13]. Several objective measurements such as dried blood spot (DBS) and other pharmacological assays have been used for measuring drug concentration in the blood, hair and urine, which are likely more accurate but costly [13, 14, 15, 16, 17]. Moreover, measuring PrEP adherence requires not only the number of pills taken but also the timing of the pills taken in relation to the timing of the sex event. With such complexity, researchers achieve limited knowledge with regard to measuring adherence from a prevention‐effective perspective in real‐world settings. Increased adoption of ED dosing regimens among MSM has been observed. In the PrEP demonstration projects, 27% to 55% of MSM PrEP users chose to take ED PrEP and 13% to 29% of PrEP users switched between daily and ED PrEP dosing regimens [18, 19, 20]. An Australian study also indicated that MSM have switched from daily PrEP to ED PrEP during the COVID‐19 pandemic [21]. These results indicate that PrEP users may switch between different dosing regimens to adapt to their sex life in real‐world settings.

It is estimated that 8.9% of MSM were on PrEP in 2019 in Taiwan [22]. The reimbursement of PrEP medication expense in Taiwan included government subsidies and user payments. MSM PrEP users in Taiwan could freely choose their PrEP dosing regimens and switch between daily and ED based on their sex life and preferences. Half of MSM PrEP users in Taiwan using ED PrEP and 8% of MSM PrEP users switched between two dosing regimens [19]. It provides a great opportunity for us to understand PrEP adherence from a prevention‐effective perspective. Hence, the purpose of this study was to describe the relationship between the use of PrEP and sex events among the MSM PrEP users. We aimed to measure PrEP adherence from a prevention‐effective perspective and identify factors associated with adherence among daily and ED MSM PrEP users enrolled in this study.

## 2 METHODS

### 2.1 Study design and participants

This study is a multicentre, observational, prospective cohort study conducted from January 2018 to December 2019 at three hospital‐based clinics in three urban cities of Taiwan with high accessibility of PrEP prescription, including Kaohsiung City, Taipei City, and Tainan City. Eligible participants for enrolment in this cohort study were HIV‐negative MSM, aged 18 years or older, at high risk of HIV acquisition, and were taking PrEP during the study period. High risk was defined as reporting one or more of the following behaviours: had condomless anal sex or sexually transmitted infections (STI) in the previous six months, were in an HIV sero‐discordant relationship, had used HIV post‐exposure prophylaxis (PEP) more than once within a previous 12 months, and had engaged in sexualized drug use [23]. This analysis included participants who provided written informed consent, were enrolled between 1 January 2018 and 15 December 2019, and had baseline data. At each visit, participants received detailed information and counselling about two PrEP dosing regimens (daily and ED). Participants were advised to return for laboratory follow‐up and prescription refills at least every three months. A detailed description of study procedures has been published previously [19]. Data on sexual behaviours and PrEP dosing regimen choices were collected through a self‐administrated questionnaire at each visit. This study was reviewed and approved by the Institutional Review Board of the National Cheng Kung University Hospital [#B‐BR‐106‐046].

### 2.2 Measurement

#### 2.2.1 PrEP dosing and adherence

Adherence was defined as taking PrEP correctly. Participants reported details of their PrEP use within five days around the most recent anal intercourse in the past month at each visit. A correct intake of PrEP for the most recent anal intercourse in the past month was defined as follows: (1) for ED regimen, taking two pills on the day having sex or one day before sex, and followed by two single doses on the following two days after the day first drug intake; for example taking two pills on day X (i.e., the day having sex) and one pill a day from the day X + 1 to the day X + 2; (2) for daily regimen, at least one pill a day from day X − 2 to day X + 2 [20]. Missed doses with ED regimen were further categorized as pre‐coital, post‐coital and both.

#### 2.2.2 Sociodemographic characteristics, sexual behaviour, substance use and mental health

Age, gender, sexual orientation, educational level, monthly income and relationship status were collected at baseline. At baseline and each follow‐up visit, participants were asked about their sexual behaviours. The number of anal sex partners in the past six months at baseline and the past month for each follow‐up was divided into four categories: no person, one person, two to five persons, and more than five persons. The number of condomless anal sex episodes in the past four weeks at baseline and follow‐ups was also collected as a continuous number. A history of PEP use in the last year was only collected at baseline. Data on substance use in the past year at baseline and the past month at the follow‐up were collected, including the following: alcohol and other recreational drugs use (MDMA, ketamine, GHB/GBL, methamphetamine and mephedrone), and acquisition of STI in the lifetime at the baseline and in the last three months at the follow‐up.

Anxiety and depression were measured with seven items on the Generalized Anxiety Disorder (GAD‐7) scale and the Patient Health Questionnaire‐9 (PHQ‐9) that were assessed for a period of two weeks. GAD‐7; PHQ‐9 questionnaires were collected at baseline and at each visit. Both scales used a 4‐point scale, with scores ranging from 0 (rarely) to 3 (most of the time). We divided both GAD‐7 and PHQ‐9 results into two categories: cutoff at 10 for GAD‐7 and at 15 for PHQ‐9 [24, 25].

#### 2.2.3 PrEP‐related behaviours

Data on PrEP‐related behaviours were collected at each visit. Self‐identified PrEP dosing regimens were divided into three categories: daily, ED, and mixed. We combined ED and mixed for the analysis. A PrEP dosing regimen switch was defined by inconsistent PrEP dosing between the current and the last visit. PrEP dose switching was classified into four categories: daily and no switch, ED and no switch, switched from daily to ED and switched from ED to daily.

#### 2.2.4 Follow‐up time calculation

End of follow‐up was defined as the cut‐off date of this analysis. For patients with HIV seroconversion, we defined the midpoint between the date of last HIV‐test and the date of first positive test as the point of HIV seroconversion. If participants decided to leave this study, the date they claimed to drop out would be the time for censoring.

### 2.3 Statistical analysis

We performed all statistical analyses using SAS 9.4 (SAS Institute Inc., Cary, NC, USA). Demographic characteristics at baseline were presented as median with an interquartile range for continuous variables and as the proportion for categorical variables. The HIV incidence rate was calculated as the total number of HIV infections divided by person‐years of observation. We calculated 95% CIs for incidence rate using exact Poisson methods.

We examined the number of pills taken and the sexual risk behaviours between daily and ED PrEP dosing regimens. To assess the pills taken between regimens by each participant over time, a mixed effect linear regression model was fitted with participant‐specific random intercept, the indicator for daily versus ED regimens. A similar mixed effects Poisson regression model was used to assess the number of condomless anal intercourse episodes’ overtime. The number of anal sex partners for daily and ED PrEP dosing regimens was fitted with a mixed effect multinomial regression model.

Univariable logistic mixed‐effect models were first used to identify factors associated with PrEP adherence. Factors that were significant at *p* ≤ 0.05 level in these models were considered for inclusion in the final multivariable model. In the final multivariable model, we further adjusted for time since PrEP initiation. We implemented two multivariable logistic mixed‐effect models (Model 1 and Model 2) to assess factors associated with PrEP adherence. Model 1 assesses the association between PrEP adherence and PrEP dosing regimens. In Model 2, we assessed the association between PrEP adherence and dose switching. In Model 2, we only included those participants who had data from more than one visit.

## 3 RESULTS

### 3.1 Study participants

From 1 January 2018 to 15 December 2019, there were 374 MSM participants included in the study, with 1,054 visits. Half MSM were under 30 years of age (51.6%). A total of 39.9% of participants had STI and 18.2% used sexualized drugs in the past year (Table 1.).

**Table 1:** Baseline characteristics of PrEP users (N = 374)

	PrEP users (N = 374)
	N (%)^a^
Age, years	
≤30	193 (51.6)
31 to 40	137 (36.6)
>40	44 (11.8)
Study Site	
Integrated sexual health clinics	280 (74.9)
Medical centre	94 (25.1)
Education	
Master degree or higher	104 (27.8)
Below Master degree	270 (72.2)
Salary (per month)	
>30,000 NTD	242 (64.7)
≤30,000 NTD	132 (35.3)
GAD‐7 score	
Moderate or severe anxiety (≥10)	37 (9.9)
Minimal or mild anxiety (<10)	337 (90.1)
PHQ‐9 score	
Moderately severe or severe depression (≥15)	13 (3.5)
Moderate, mild, or no depression (<15)	361 (96.5)
Currently in a relationship^b^	100 (27.8)
Self‐reported STI in the lifetime (any kind)	146 (39.9)
Alcohol use in the past year^c^	71 (19.4)
Recreational drug use in the past year^*^	68 (18.2)
PEP use in the past year^d^	55 (15.2)
Number of total sex partners in the last 6 months^e^	
0	1 (0.3)
1	46 (13.4)
2 to 5	174 (51.6)
>5	117 (34.7)
Insertive condomless anal intercourse in the last six months^e^	210 (62.3)
Receptive condomless anal intercourse in the last six months^e^	223 (66.2)
Number of condomless anal sex episodes in the past four weeks^f^	
Median [IQR]	1 [0 to 3]
<5	233 (86.3)
≥5	31 (11.7)
Had PrEP use experience^g^	125 (38.7)

1 US Dollars, 30 New Taiwan Dollars; GAD‐7, generalized anxiety disorder 7; NTD, New Taiwan Dollars; PEP, Post‐Exposure Prophylaxis; PHQ‐9, patient health questionnaire‐9; STI, sexually transmitted infections.

^a^ Not all percentages add up to 100% due to rounding

^b^ 14 missing

^c^ 1 missing

^d^ 12 missing

^f^ 110 missing

^g^ 51 missing

**Figure 1:**
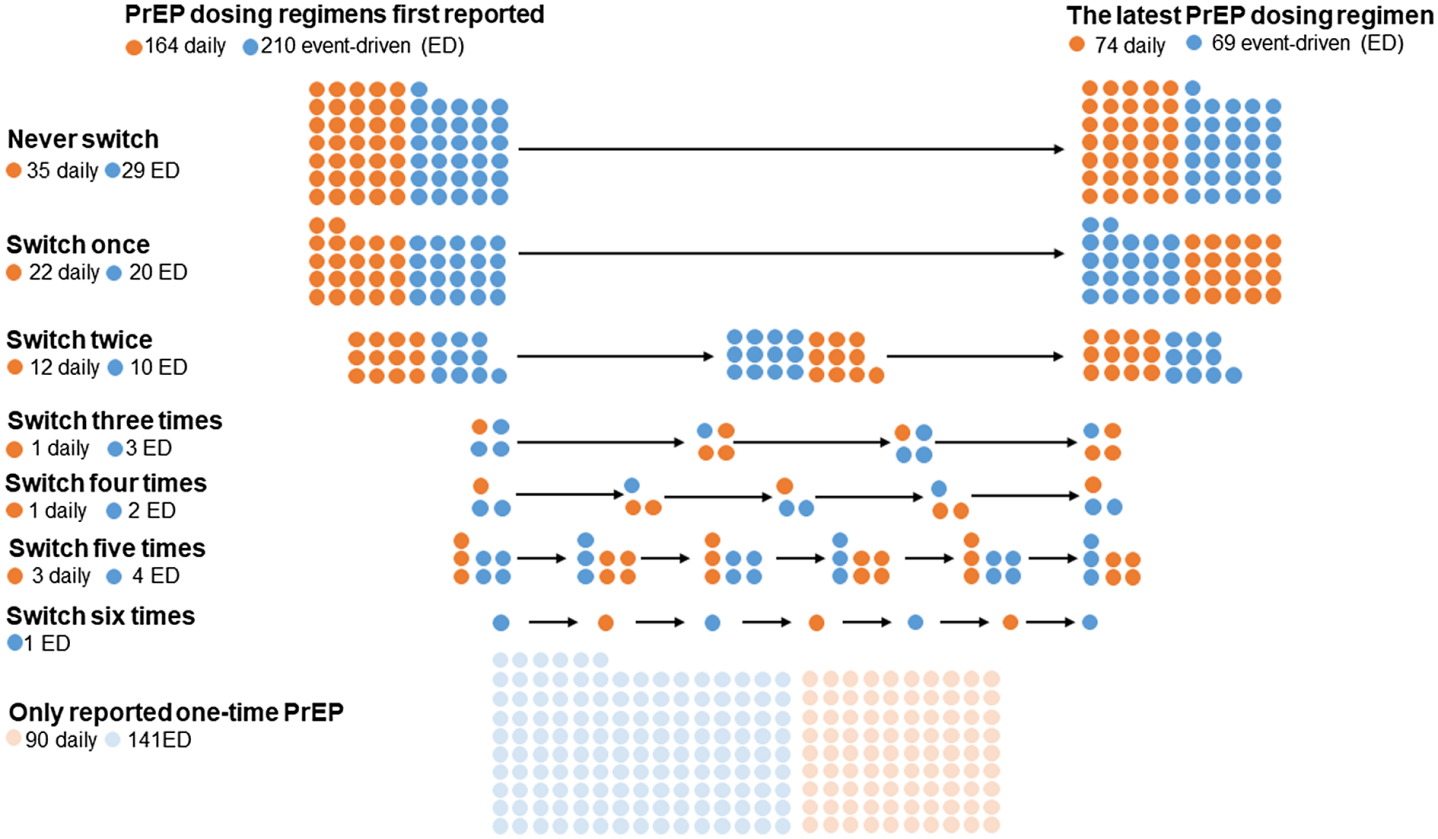
Pattern of PrEP dosing switching (N = 374).

Figure 1 depicts participants’ initial choices and their switching frequency of PrEP during the study period. Two‐hundred and ten participants (56%) reported taking PrEP ED and 164 (44%) reported daily PrEP use at enrolment. Seventy‐eight participants (20.9%) reported 150 regimen switches: 74 from daily to ED and 76 from ED to daily, which was 54.5% (78/143) of reported PrEP use in the past month in more than one visit. Eight participants (2%) had switched their dosing regimens five times or more. Sixty‐eight participants (48.3%) were on ED PrEP for their latest visit; 74 participants (51.7%) were on daily PrEP. A total of 69.7% of participants received government‐subsidized PrEP medication in 74.7% of visits (787/1054 visit). The total follow‐up time was 154 person‐years (median of follow‐up days: 106 days, IQR: 35 to 231 days).

The overall HIV incidence was 0.69 per 100 person‐years; the 95% CI was 0.098 to 4.852. One participant acquired HIV during the follow‐up. The participant took daily PrEP and never switched to ED PrEP during the follow‐up. The participant had been on daily PrEP for four months. He engaged in condomless anal intercourse with a HIV positive and viral load‐detectable sexual partner. Self‐reported PrEP adherence was high; however, he missed a few pills a month prior to when his HIV sero‐conversion was detected. One missed pill was within the five days of condomless anal intercourses with an HIV positive and viral load‐detectable sexual partner. The participant was referred to an infectious disease clinic in a medical centre for further management of his HIV infection.

### 3.2 Pills taken in the past month comparing two dosing regimens

**Figure 2:**
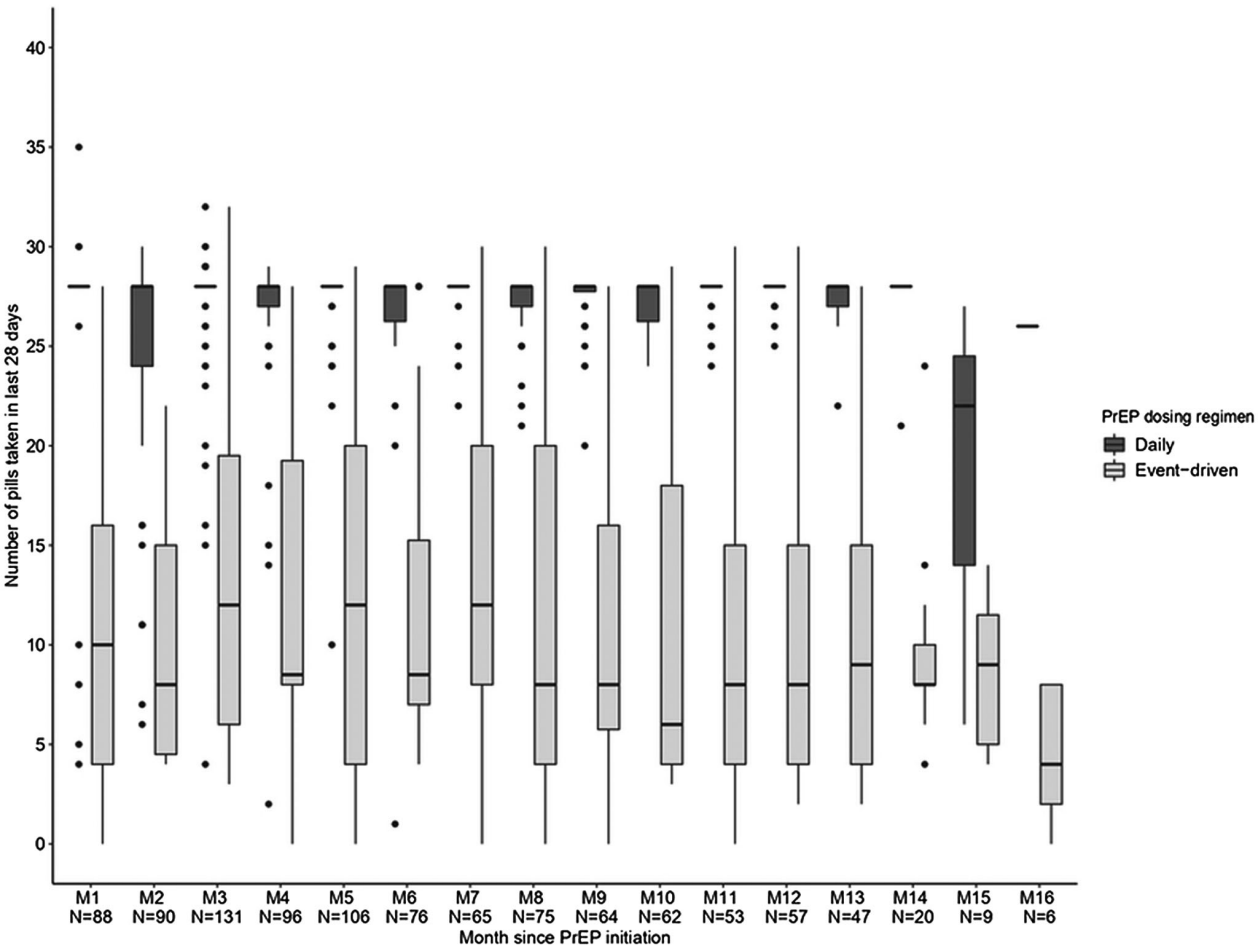
Boxplot of the number of pills taken in last 28 days comparing two dosing regimens. Number of participants in each month may not sum precisely to total number of participants (N = 374) due to missing data. The dots in the figure were the outliners of number of pills taken in the last 28 days.

The number of pills taken in the past 28 days in different PrEP dosing regimens over time is shown in Figure 2. The number of pills taken in the past 28 days was significantly higher when participants reported daily PrEP use (27, 95% CI: 26 to 28) than ED PrEP use (12, 95% CI: 11 to 13) (mixed effect linear regression model, *p*‐value <0.001).

### 3.3 Sexual risk behaviours in the past month comparing two dosing regimens

**Figure 3:**
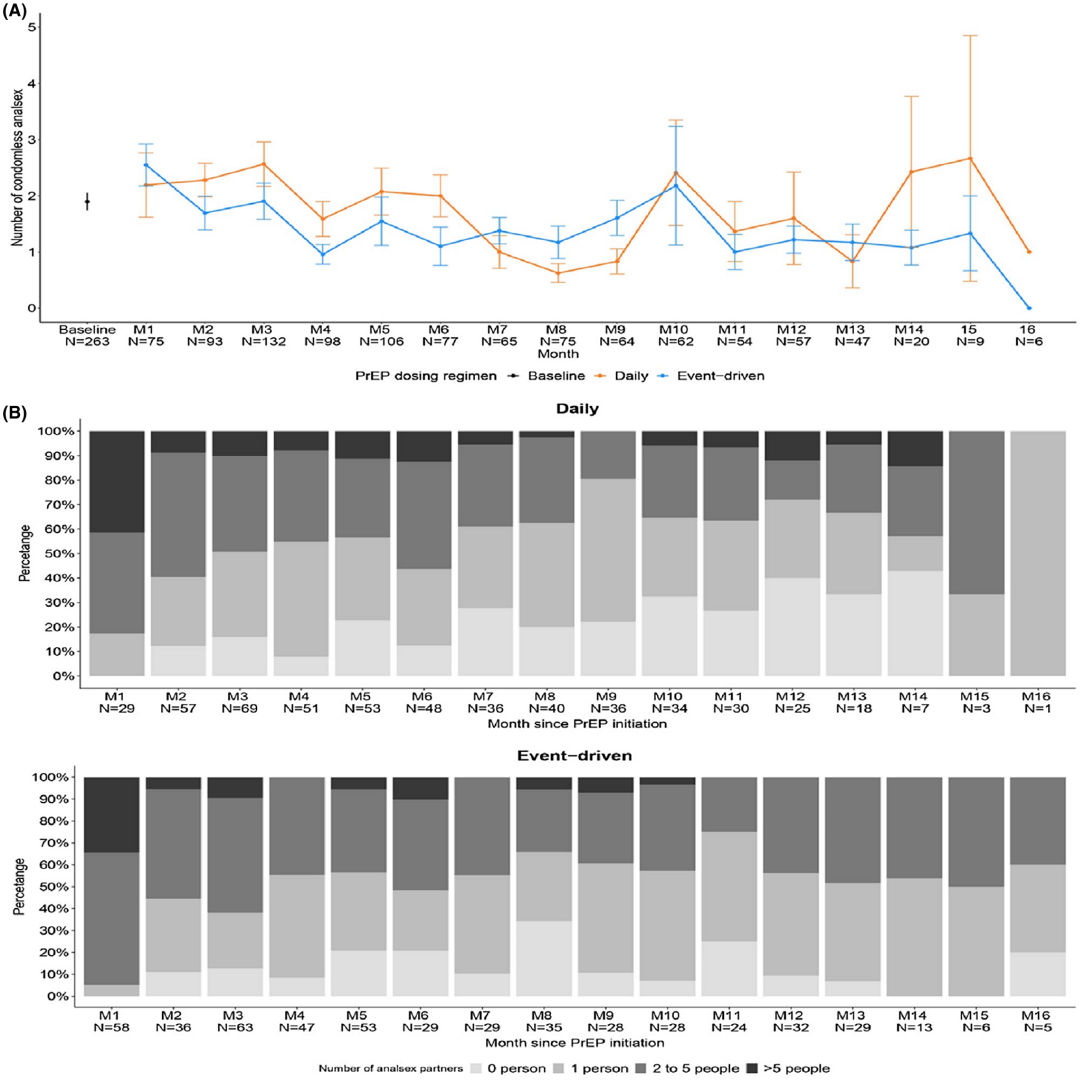
Sexual risk behaviour comparing two dosing regimens over time. (A) The number of condomless anal sex overtime per dosing regimen. (B) The number of anal sex partners over time per dosing regimen. Number of participants in each month may not sum precisely to total number of participants (N = 374) due to missing data

Figure 3 shows the sexual risk behaviours between two dosing regimens over time.

The number of instances of condomless anal sex had no significant difference between daily and ED dosing regimen and did not change significantly over the study period in both groups (mixed effect Poisson regression) shown in Figure 3A. Similarly, the number of anal sex partners did not differ significantly between two dosing regimens (mixed effect multinomial regression model) shown in Figure 3B.

### 3.4 Pattern of adherence among PrEP users

Overall, PrEP was taken correctly in 84.2% of visits regardless of dosing regimen (86.6% in daily dosing vs. 81.8% in ED dosing). For those visits in which participants reported non‐adhering to ED PrEP use (94 visits), 39 visits (41.5%) were taking ED PrEP and never switched, 21 visits (22.3%) were switched daily to ED PrEP and 34 visits (36.2%) were on ED PrEP but had only one visit. The missing pattern was that 81.9% missed only pre‐coital doses, 4.3% missed only post‐coital doses and 13.8% missed both pre‐ and post‐coital doses (Table 2.).

**Table 2:** Pattern of non‐adhered timing among event‐driven PrEP users (94 visits)

	ED, never switch (n = 39)	Daily to ED (n = 21)	ED, only one visit (n = 34)	Total visits (n = 94)
Only pre‐coital	34 (87.2%)	18 (85.7%)	25 (73.5%)	77 (81.9%)
Only post‐coital	1 (2.6%)	0 (0%)	3 (8.8%)	4 (4.3%)
Both	4 (10.3%)	3 (14.3%)	6 (17.7%)	13 (13.8%)

### 3.5 Factors associated with PrEP adherence

In the univariable logistic mixed‐effect model, PrEP adherence was negatively associated with participants who showed anxiety and depression, who engaged in sexualized drug use and who took PrEP ED. PrEP adherence was significantly associated with having two to five sexual partners in the last month, compared to participants who had no sexual partners in the last month.

In multivariable Model 1, having two to five sexual partners in the last month (aOR = 2.07, 95% CI: 1.18 to 3.62), sexualized drug use (OR = 0.50, 95% CI: 0.27 to 0.94) and taking PrEP ED (aOR = 0.54, 95% CI: 0.36 to 0.81) remained significantly associated with taking PrEP correctly, after controlling for the time since follow‐up.

In Model 2, switching from daily to an ED regimen was negatively associated with PrEP adherence (aOR: 0.30, 95% CI: 0.15 to 0.63) compared to a daily dosing regimen and no switching from the last visit. Yet, the odds of taking PrEP ED and no switch (aOR: 0.56, 95% CI: 0.30 to 1.06) and switching from ED to daily (aOR: 0.52, 95% CI: 0.24 to 1.15) did not have a significant difference compared to a daily dosing regimen and no switch from the last visit (Table 3.)

**Table 3:** Factors associated with PrEP adherence using mixed effect logistic model

	Univariable		Multivariable			
			Model 1 (1,054 observations)		Model 2 (680 observations)	
	OR (95% CI)	*p*‐value	aOR (95% CI)	*p*‐value	aOR (95% CI)	*p*‐value
Baseline characteristics						
Age (ref ≤30)	1.02 (0.99 to 1.06)	0.214				
In stable relationship	0.69 (0.47 to 1.02)	0.061				
Education (Above college vs. college graduate or below)	1.57 (0.96 to 2.56)	0.074				
Salary (≥1,000 USD vs. <1,000 USD)	1.07 (0.72 to 1.60)	0.728				
PEP in the past year	1.64 (0.86 to 3.12)	0.130				
Time‐varying variables^a^						
Time (days)^b^			1.00 (1.00 to 1.00)	0.615	1.00 (1.00 to 1.02)	0.983
Anxiety (≥10 vs. <10)^c^	0.46(0.24 to 0.90)	0.023*	0.70 (0.30 to 1.67)	0.422	0.58 (0.18 to 1.88)	0.364
Depression (≥15 vs. <15)^d^	0.35 (0.14 to 0.87)	0.025*	0.58 (0.17 to 1.96)	0.379	0.55 (0.11 to 2.64)	0.453
Had STI	0.76 (0.46 to 1.26)	0.286				
Number of anal sex partners in the past month (ref = 0)						
1	1.41 (0.83 to 2.40)	0.206	1.40 (0.82 to 2.41)	0.222	1.49 (0.75 to 2.96)	0.258
2 to 5	1.84 (1.07 to 3.17)	0.027*	2.07 (1.18 to 3.62)	0.011*	2.02 (0.98 to 4.15)	0.056
>5	0.70 (0.34 to 1.41)	0.317	0.84 (0.40 to 1.77)	0.653	0.78 (0.30 to 2.06)	0.617
Number of CAS in the past 4 weeks (≥5 vs. <5)^e^	0.58 (0.32 to 1.05)	0.072				
Alcohol use in the past month	0.92 (0.51 to 1.67)	0.782				
Sexualized drug use in the past month^f^	0.50 (0.28 to 0.89)	0.018*	0.50 (0.27 to 0.94)	0.031*	0.62 (0.25 to 1.50)	0.286
PrEP dosing regimen (Event‐driven vs. daily)	0.58 (0.39 to 0.86)	0.007**	0.54 (0.36 to 0.81)	0.003**		
Dosing regimens switch (ref= daily, no switch)						
Event‐driven, no switch	0.61 (0.32 to −1.13)	0.116			0.56 (0.30 to 1.06)	0.071
Daily to event‐driven	0.31 (0.15 to 0.63)	0.001***			0.30 (0.15 to 0.63)	0.001***
Event‐driven to daily	0.47 (0.22 to 1.02)	0.056			0.52 (0.24 to 1.15)	0.105

Model 2 only included those participants who had data from more than one visit with 680 observations. The variables were the same as Model 1 except that Model 2 included dosing regimen switching rather than PrEP dosing regimen. AOR, adjusted odds ration; CAS, condomless anal sex; OR=odds ration; PEP, Post‐exposure Prophylaxis; STI, sexually transmitted infections.

^a^ Adjusted time since first self‐report PrEP initiation during the study period in univariate model

^b^ time: Since first self‐report PrEP initiation during the study period, unit: month

^c^ generalized anxiety disorder 7‐item (GAD‐7) scale score

^d^ patient health questionnaire (PHQ‐9) scale score

^e^ CAS: condomless anal sex

^f^ defined as use of methamphetamine or ecstasy or GHB/GBL or ketamine or mephedrone.

* odds ratio significant at the *p* ≤ 0.05 level

** odds ratio significant at the *p* ≤ 0.01 level

*** odds ratio significant at the *p* ≤ 0.001 level.

## 4 DISCUSSIONS

Our study in this multicentre cohort of Taiwanese MSM indicates that PrEP was taken correctly during 80% of the most recent anal intercourse episodes. Switching from daily to ED use was associated with a greater likelihood of taking PrEP incorrectly compared to those participants who reported daily use and no switch. The flexibility of choosing daily or ED PrEP among MSM PrEP users in Taiwan provided a unique opportunity to explore factors associated with PrEP adherence in a real‐world setting.

A major finding of this study was that we identified that switching from daily to ED was associated with an increased rate of taking PrEP incorrectly for the most recent anal intercourse in the past month. During the follow‐up period, we observed a substantial proportion of MSM PrEP users switching dosing between daily and ED PrEP during the follow‐up period.

Similar conditions were found in studies from European countries that also allowed participants self‐selection and participants could switch between daily and ED PrEP, according to their preferences. The Be‐PreP‐ared study in Belgium found that 19% of participants switched their PrEP dosing regimens, similar to our study [20]. The AMPrEP study in the Netherlands found a higher proportion of participants (30%) who had switched regimens during a two‐year follow‐up period [18]. These findings suggest that PrEP users might adopt PrEP dosing regimens according to their lifestyle over time, rather than restrict themselves to a single dosing regimen. The complexity of PrEP adherence increases for MSM switching between two dosing regimens based on their sex lives or preferences and the complexity of PrEP adherence measurement also increased in real‐world settings.

Studies have indicated that ED PrEP may result in lower adherence than daily use [26, 27]. Without considering switching, ED PrEP users were less likely to adhere to PrEP use than daily PrEP users. PrEP adherence in our study, however, was not significantly different between participants who never switched dosing regardless of their initial dosing choices. Instead, participants switching from daily to an ED dosing regimen were less likely than daily users to adhere to PrEP and users who never switched. This finding is compatible with the assumption that ED PrEP is more complex and technically demanding dosing than daily, since the users need to be able to plan sexual intercourse in advance to take a pre‐coital dose and follow more stringent and less forgiving post‐coital doses. Although being a relatively more complex dosing regimen, ED PrEP may be familiarized by users by repeating the regimen. This may be one of the reasons we see no significant differences between ED users who never switched and daily users who never switched.

Our results suggest that maintaining sufficient PrEP coverage of at‐risk sexual intercourses could be challenging for MSM PrEP users. This highlights the need for novel interventions to assist PrEP users in self‐monitoring their PrEP adherence, to guide the users with correct PrEP dosing according to their sex events, and even more important to allow PrEP users to switch their dosing regimens safely.

How ED PrEP users missed their doses has not been reported in the literature. Our results found that participants who took ED PrEP were more likely to miss doses before sex compared to missing doses after sex in real‐world settings. Studies that compared the missed doses between daily, intermittent, and ED PrEP use have indicated that post‐coital doses for ED PrEP use were more likely to be missed than daily or intermittent dosing [28]. However, it should be noted that the studies compared missed doses between dosing regimens, whereas our study focused on missed doses within ED PrEP use. Our study implied that not all participants choosing ED PrEP were capable of planning sexual intercourse in advance.

HIV incidence was low in our cohort and no ED PrEP users acquired HIV during the follow‐up; however, PrEP was taken incorrectly during the most recent anal intercourse in a substantial portion (15.8%) of the visits. From a prevention‐effective adherence perspective, PrEP adherence – where adhering to PrEP perfectly might not be essential – needs to be considered with other HIV prevention strategies, such as using condoms, or having sex with someone on PrEP or having an undetectable HIV viral load [8, 10, 29, 30, 31].

Recognizing the risk of HIV exposure in coordination with planning sex in advance is challenging for PrEP users in practice. PrEP adherence counselling should help PrEP users understand and accurately assess their risk of HIV and make appropriate decisions about prevention options.

Our study has several limitations. First, we only collected PrEP intake within five days around the most recent anal intercourse and dosing regimen in the past month at each visit to avoid recall biases. However, this might not be able to depict the full pattern of dosing regimen switch and adherence among PrEP users over time because between two visits, there might be other switches that were not reported. Switching behaviours might be under‐reported.

Second, a high proportion of participants reported only one‐time point of PrEP use in our study. We only included participants who had data from more than one visit in Model 2 to explore the association between adherence and dose switching. This may result in bias in generalizability. Our study results may only be applicable to those who regularly return to clinics, but not to those who rarely come back for follow‐ups.

Third, for taking 2 pills, we did not assess whether these 2 pills were taken two to twenty‐four hours before sex because we did not ask participants the accurate timing for taking pills. It is possible that participants might have not taken the pills two to twenty‐four hours before sex, but we may have mistaken it as correct doses. Hence, we might overestimate the proportion of correct use for ED PrEP.

Last, we did not collect data on the objective measures of their risk of HIV exposure such as condom use, HIV status and viral load of their sex partners. From prevention‐effective adherence, the use of other effective HIV prevention tools should be considered for estimating the risk of HIV exposure. However, communication regarding HIV status is still a sensitive subject that most MSM do not want to discuss with their sex partners. Studies have shown that 39% of MSM PrEP users had at least one sex partner with unknown HIV status [32], and did not know their sex partners’ HIV viral load or whether their sex partners were taking PrEP [33, 34, 35]. Considering that estimating the risk of HIV exposure is challenging [36], we deem anal intercourse as a risk of HIV exposure regardless of condom use or effective HIV prevention strategies use during sex. In the future, measurement of PrEP adherence should incorporate more details regarding the sex event, such PrEP adhering patterns and other HIV prevention strategies, which mandate innovative, real‐time HIV preventive approaches.

## 5 CONCLUSIONS

In conclusion, our study demonstrated that a high level of PrEP adherence was observed in the majority of MSM who returned to PrEP services. Switching from a daily to an ED dosing regimen was found to be more challenging to PrEP adherence. Innovative approaches that obtain the context of PrEP adherence should be tailored specifically to MSM PrEP users to better understand PrEP adherence in real‐world settings, especially for those who switched from daily to ED PrEP.

## COMPETING INTERESTS

The authors have no competing interests to declare.

## AUTHORS’ CONTRIBUTIONS

HJW analysed the data. HJW, CS wrote the paper. HJW, SWWK, CWL, HHC, NYK, CS contributed essential reagents and revised the paper. CS designed the research study.
